# Development of reading fluency and metacognitive knowledge of reading strategies during reciprocal teaching: do these changes actually contribute to reading comprehension?

**DOI:** 10.3389/fpsyg.2023.1191103

**Published:** 2023-08-24

**Authors:** Maris Juhkam, Anna-Liisa Jõgi, Piret Soodla, Mikko Aro

**Affiliations:** ^1^Tallinn University, Tallinn, Estonia; ^2^University of Jyväskylä, Jyväskylä, Finland

**Keywords:** reading fluency, reading strategies, reading comprehension, reciprocal teaching, learning difficulties

## Abstract

The study examined the effect of reciprocal teaching on students’ reading comprehension, reading fluency, and metacognitive knowledge of reading strategies, especially among students with learning difficulties. The special focus was to assess how the increase in reading fluency and metacognitive knowledge during the intervention contributes to the reading comprehension outcome. The sample consisted of 301 Grade 3 students, of whom 77 had learning difficulties. Analyzes of (co)variances were used for estimating the effects of reciprocal teaching on the development of reading comprehension, reading fluency, and metacognitive knowledge. Multigroup path analysis was used for testing the effect of increase in reading fluency and metacognitive knowledge on reading comprehension. We found reciprocal teaching had a positive effect on reading comprehension, especially for students with learning difficulties. Reading fluency and metacognitive knowledge improved significantly, but similarly in both the intervention and control groups. However, the increase in metacognitive knowledge only contributed to reading comprehension in the intervention group, not in the control group. The study sheds light on the cognitive and metacognitive mechanisms underlying students’ reading comprehension process, emphasizing metacognitive knowledge and especially the systematic practice of reading strategies as key factors in improving reading comprehension.

## Introduction

Reading comprehension (RC) has long been considered the basis for acquiring academic education (Mastropieri and Scruggs, 1997). However, in many countries, a significant number of secondary school graduates have lower-than-expected levels of RC, meaning they have difficulty understanding even elementary-grade-level texts (OECD, 2016, 2019). Although certainly concerning, this finding is not really surprising as RC is a cognitively demanding task, requiring both cognitive and metacognitive skills (Cain et al., 2004; Cain and Oakhill, 2006; Hannon and Frias, 2012). Furthermore, RC is especially difficult for students with learning difficulties (LD), who–in addition to lower levels of cognitive skills–often have attention difficulties and motivational problems (Kendeou et al., 2014; Berkeley and Larsen, 2018) as well as insufficient metacognitive knowledge and skills (Wigent, 2013; Bergey et al., 2017).

Thus, to support the acquisition of academic education, a number of intervention studies have been conducted to find ways to effectively improve students’ RC (Alnahdi, 2015; Wright and Cervetti, 2017; Stevens et al., 2019; Silva-Maceda and Camarillo-Salazar, 2021; Serrano-Mendizabal et al., 2023). One particular focus in the meta-analyzes are interventions teaching the use of reading strategies (Rosenshine and Meister, 1994; Sencibaugh, 2007), thereby improving readers’ metacognitive knowledge and skills to become strategic readers. In studies focused on teaching reading strategies, intervention effects have mostly been reported separately on RC and metacognitive knowledge of reading strategies (Muijselaar et al., 2018; Gu and Lau, 2021; Wu et al., 2021), although sometimes studies have also looked at reading fluency as a prerequisite of RC (Ritchey et al., 2017). However, a closer look reveals that sometimes interventions focused on teaching reading strategies only had an effect on metacognitive knowledge of reading strategies, not on RC (e.g., Ritchey et al., 2017; Muijselaar et al., 2018). Thus, the question remains regarding the mechanism of such interventions–namely, how and to what extent the teaching of reading strategies actually contributes to the development of RC. As far as we know, the only exception that attempts to reveal the mentioned mechanism is the study by Spörer and Schünemann (2014), which investigated the direct contribution of the growth of metacognitive knowledge of reading strategies during Reciprocal Teaching method (RT; Palincsar and Brown, 1984) to RC outcome. Meanwhile, changes in reading fluency and the contribution of reading fluency growth to RC within interventions focused on teaching reading strategies have remained unexplored.

In the case of the RT method, it is particularly surprising that it has not been thoroughly investigated whether and to what extent the increase in metacognitive knowledge of reading strategies and the increase in reading fluency during the intervention directly contribute to RC, because RT method is developed already decades ago (Palincsar and Brown, 1984) and it is a well-known and widely studied method (e.g., see review study by Rosenshine and Meister, 1994; Spörer et al., 2009; Spörer and Schünemann, 2014; Tseng and Yeh, 2018; Thurston et al., 2020). The RT method is based on three principles: (1) the conscious use of four comprehension-fostering strategies is practiced while reading (predicting, clarifying, questioning and summarizing); (2) during the process, students are actively supported through expert modeling of strategies, i.e., explicit instruction is used; (3) students practice using strategies in groups, supporting and guiding each other. Students with LD, who have particular difficulties with RC due to their lower cognitive and metacognitive knowledge and skills (Wigent, 2013; Bergey et al., 2017; Berkeley and Larsen, 2018), have also been found to benefit from the RT method (see systematic review study by Lee and Tsai, 2017). Based on the principles of inclusive education, students with LD are increasingly included in regular classes. Therefore it is important to investigate even more thoroughly how the RT intervention carried out in the classroom, where students with LD study alongside students with age-appropriate development, supports the development of students with LD.

The current study aims to investigate the extent to which the increase in reading fluency and metacognitive knowledge of reading strategies during RT intervention (Palincsar and Brown, 1984) predicts RC by the end of the intervention. We chose the RT method to conduct our study because, on the one hand, it is one of the most studied and widely used method for teaching reading strategies and thereby developing RC. On the other hand, only little is known even about this widely used method, whether and to what extent the growth of metacognitive knowledge of reading strategies and the growth of reading fluency during the implementation of the RT method actually contributes to the RC. The study provides important information about the development of cognitive (specifically about reading fluency) and metacognitive (the knowledge about reading strategies) processes that support RC through the method of RT, and thereby helping to implement the intervention in a more targeted manner in pedagogical practice. The specific focus of our research is also on the intervention effect on students with LD, who often need particular support in the development of RC. At the same time, it is crucial to emphasize that RC, in turn, is the basis for the acquisition of academic education as well as for success later in life (Catts and Kamhi, 2017).

### Role of reading fluency and metacognition in reading comprehension

The simple view of reading (SVR) defines RC as an outcome of language comprehension and decoding skills. Linguistic comprehension is the ability to process and understand orally presented words, sentences, and texts while decoding refers to the ability to read words quickly and accurately (Gough and Tunmer, 1986; Hoover and Gough, 1990). Decoding skill proceeds from learning the correspondences between phonemes and graphemes to the ability to use these correspondences in reading syllables and words and finally being able to read the words quickly and accurately using letter patterns as stored units (Frith, 1986; Ehri, 2005). As basic decoding skills become more efficient and automated, the fluency of reading increases, meaning the reader is able to read connected text rapidly, accurately, and with appropriate prosody (Fuchs et al., 2001; Veenendaal et al., 2015). With the ability to read text fluently, the reader no longer has to pay attention to decoding and thus can allocate more resources to the main purpose of reading: reading comprehension (Perfetti, 2007). Several studies have shown a link between reading fluency and RC, which is stronger in early stages of reading acquisition (e.g., Kim et al., 2011, 2012) and exists irrespective of the depth of the orthography (Florit and Cain, 2011). A recent literature review revealed that interventions aimed at developing reading fluency (most often the repeated reading method) positively affect not only the speed and accuracy of reading, but also, in some cases, RC (Steinle et al., 2022).

In addition to linguistic comprehension and decoding skills, metacognition is becoming increasingly important as reading skills develop and texts become more complex. Metacognition is generally defined as the ability to monitor one’s learning process, notice when learning is no longer effective (for example, difficulties arise in understanding the meaning of the read text), and use effective strategies to ensure success in the learning process and outcomes (Alexander, 2008). Thus, teaching the use of metacognitive strategies is key for developing self-regulated learners who actively take responsibility for their own learning (Rovers et al., 2019; Wirth et al., 2020). Perry et al. (2019) found in their literature review strong evidence that effective teaching of metacognition in school is associated with academic success. According to Zimmerman’s (1998) model of self-regulated learning, the most important thing in learning is not the product of learning, but the awareness of the process. In his model, Zimmerman (1998) distinguishes three aspects that are important to follow in the learning process: (a) goals setting and strategic planning, (b) self-monitoring of one’s learning process and implementation of strategies, (c) self-assessment of strategy outcome and task performance.

The importance of metacognition is also increasingly emphasized in the context of language learning, the primary input and basis of which is listening (including vocabulary, morphology and syntax; e.g., Chamot, 2005). Interventions focused on teaching metacognitive strategies–more specifically, listening strategies–have been found to be effective in developing listening comprehension (Veenman et al., 2006; Bozorgian et al., 2022), which is crucial for RC (Gough and Tunmer, 1986; Hoover and Gough, 1990). In several of his interventions, Bozorgian (2014) and Bozorgian et al. (2022) has used Vandergrift (2004) “pedagogical cycle” framework, a process-based approach to developing listening comprehension. This framework includes both listening instruction, which directs students to become aware of the listening process, and metacognitive elements, such as planning, self-monitoring, self-evaluation. Thus, the principle of developing metacognition in the Vandergrift (2004) cycle is very similar to Zimmerman’s (1998) theory of self-regulated learning.

When addressing metacognition in the context of reading, the term *reading strategies* is often used, which is defined as goal-directed attempts that support the construction of the meaning of a text as well as the monitoring and guiding of one’s own RC process (Andreassen and Bråten, 2011; Afflerbach et al., 2020). Many reading strategies support RC, such as activating prior knowledge before reading, overviewing before reading, asking questions about oneself while reading, underlining important information, and summarizing (Pressley, 2002; Afflerbach et al., 2020). However, in the context of using reading strategies, it is important to distinguish between *metacognitive knowledge* (i.e., the reader knows which strategies effectively support RC, such as what to do if a sentence is not understood) and *metacognitive skills* (i.e., the reader actually uses an effective strategy when the need arises during reading, such as re-reading a sentence that was not understood; Veenman et al., 2005, 2006). Metacognitive knowledge is a prerequisite for the use of this knowledge, but does not yet guarantee its use (Veenman et al., 2006). Readers may know and even use the same metacognitive strategies (e.g., some reading strategies), but the effectiveness of their use can vary greatly (Grabe, 2009). These differences may be due to the fact that the effective use of reading strategies, as metacognitive activities in general, requires the knowledge of strategies themselves and what they are needed for (declarative knowledge, which is a prerequisite for their use), knowledge of how to implement strategies effectively (procedural knowledge), and knowledge of which strategies are appropriate in the given context [i.e., reading and comprehending the given text (conditional knowledge); Flavell, 1979; Brown, 1987]. Students’ knowledge at different levels may be uneven or some knowledge may be missing completely; thus, the use of strategies cannot be effective. To develop the ability to use reading strategies effectively, the implementation of reading strategies must be taught explicitly and step by step (Pressley and Gaskins, 2006).

### Effects of interventions aimed at teaching the use of reading strategies

Several recent studies (Schünemann et al., 2013; Gu and Lau, 2021; Wu et al., 2021) as well as previous literature reviews (e.g., Rosenshine and Meister, 1994) have described the positive effects of interventions focused on teaching reading strategies on RC among students with age-appropriate academic skills as well as among students with reading or learning difficulties (Sencibaugh, 2007; Lee and Tsai, 2017). In addition to improving RC, the effect of the intervention on the development of metacognitive knowledge of reading strategies, as a separate aspect, has often been evaluated. Usually, as can be expected, a positive intervention effect has been found on the development of both RC and metacognitive knowledge (e.g., Gu and Lau, 2021; Wu et al., 2021). Nevertheless, some studies have found positive effects only on metacognitive knowledge of reading strategies, but not on RC (Ritchey et al., 2017; Muijselaar et al., 2018). Several explanations are possible for this somewhat unexpected finding. For example, teachers often feel that it is difficult to induce strategic thinking in students and explicitly relate the use of strategies to RC (Duffy, 1993). Based on Brown’s (1987) approach, this finding could also be interpreted as teachers being able to provide declarative knowledge of reading strategies whereas providing procedural knowledge is more difficult. Researchers have also discussed that teachers’ interventions are not as effective as they could be (i.e., compared to researchers’ interventions) and, therefore, do not have a positive effect on students’ RC (Muijselaar et al., 2018). Another explanation might be the insufficient time resources used for explicit instruction in RC (e.g., teaching reading strategies; Houtveen and van de Grift, 2007), meaning positive effects on RC might not appear during shorter-term interventions.

Much less research has been done on the effect of interventions to teach reading strategies on reading fluency, which is understandable as the development of reading fluency is not usually the main focus of such interventions. Nevertheless, the importance of the role of reading fluency in RC has been strongly emphasized (Perfetti, 2007; Hjetland et al., 2019). Only a few studies have described the positive effect on reading fluency resulting from interventions focused on reading strategies (e.g., Ritchey et al., 2017). The positive effect on reading fluency can be explained by the fact that readers often rely on the context of the text to recognize (decode) words faster (Perfetti et al., 1979). Therefore, using strategies while reading supports RC (i.e., understanding the context), thereby ensuring more fluent reading.

So far we have quite a lot of information on the effect of interventions focused on teaching reading strategies on RC and metacognitive knowledge of reading strategies but somewhat less information about effects on reading fluency. However, knowledge is lacking about whether and to what extent an increase in metacognitive knowledge of reading strategies and reading fluency during reading strategies intervention actually contributes to the RC outcome. Mediational studies have shown that students’ reading interest positively predicted the use of reading strategies, which in turn positively predicted the outcome of RC (Van Kraayenoord and Schneider, 1999). In addition, reading interest predicted reading fluency, which in turn predicted RC (Völlinger et al., 2018). However, Pecjak et al. (2011) did not find students’ metacognitive knowledge of reading strategies predictive of RC. Furthermore, they found reading interest not being predictive of decoding speed, which was not predictive of RC.

Among intervention studies focusing on teaching reading strategies, we found only one that assessed reading strategy knowledge’s contribution to RC. Spörer and Schünemann (2014) used the RT intervention in their study, i.e., focused on teaching the conscious use of four reading strategies (predicting, clarifying, questioning and summarizing), supporting the process through explicit modeling and student group work. They formed four experimental groups, in one of which the conventional RT method was used, while in the other three groups the RT method was combined with different self-regulation procedures. Spörer and Schünemann (2014) found that the reading strategy intervention’s effect on RC was mediated through reading strategies performance. However, the finding was present only in the subgroup where the students were also trained to consciously set goals for themselves (e.g., they were asked to answer questions such as “Which strategies would you like to practice today? How many points would you like to achieve on the reading quiz?”) and later assess their achievement of the goals, i.e., in this group, students’ development was also supported through elements of self-regulated learning (Zimmerman, 1998).

To sum up, we have quite a lot of information that RT has a positive effect on metacognitive knowledge of reading strategies and also on reading comprehension (Rosenshine and Meister, 1994; Spörer et al., 2009; Tseng and Yeh, 2018; Thurston et al., 2020), but Spörer and Schünemann (2014) study is the only one to our knowledge that has also tried to evaluate the extent to which the growth of metacognitive knowledge of reading strategies through the RT method actually contributes to the RC outcome. And nothing is known about the effect of the increase in reading fluency during the intervention of RT on the RC outcome. Therefore, since RT is a frequently used method, more detailed information about the mechanism of this method is needed.

### Estonian context

The Estonian language has a transparent orthography (Jakobson et al., 2022; Juhkam et al., 2022), so Estonian children learn to read relatively quickly (Soodla et al., 2015). Like in other languages with transparent orthography (Landerl and Wimmer, 2008; Katzir et al., 2012), Estonian primary school students’ level of accuracy in reading is high but their fluency of reading is more of a concern as it takes them longer to reach a sufficient level (Soodla et al., 2015; Jakobson et al., 2022; Juhkam et al., 2022). According to the basic school curriculum, Estonian students should achieve fluent reading by the end of the third grade (Government of the Republic of Estonia, 2011). Although the reading speed and accuracy of third-grade students still develop considerably during one academic year, even in the fourth grade, almost 10% of students have insufficient basic reading skills, meaning that their reading speed is slow and they still make reading errors (Jakobson et al., 2022; Juhkam et al., 2022).

In Estonia, students with learning difficulties, including those who have problems in reading, are supported in school through a support system that includes three levels: general support, enhanced support, and special support (Government of the Republic of Estonia, 2010). The schools determine the need for general support based on teachers’ and support specialists’ (e.g., special educators) assessments and recommendations, with no official diagnosis in terms of disability or special educational needs. General support is often provided in the form of part-time special educational support, the frequency of which depends on the student’s needs, but is usually two lessons per week (Soodla et al., 2021). If a student needs more additional support, the enhanced or special support will be provided based on an out-of-school counseling team’s (e.g., psychiatrist, psychologist, speech therapist, special education teacher) decision.

## Present study

This study used reciprocal teaching (RT), originally developed by Palincsar and Brown (1984), as the intervention. Similar to Spörer and Schünemann (2014) intervention design, elements supporting the development of a self-regulated learner (i.e., goal setting, self-monitoring, self-assessment) have been added to this intervention. In the intervention, students are taught the conscious use of four comprehension-fostering reading strategies: predicting, clarifying, asking questions, and summarizing. The method emphasizes the teacher’s expertise in modeling strategies in an explicit way and using the think-aloud strategy. Another important principle is that students discuss reading strategies in a group while supporting each other and receiving supportive guidance from the teacher.

The present study had two aims: (1) to investigate the effect of RT intervention on RC, including the development of reading fluency and metacognitive knowledge of reading strategies, in students with and without LD; and (2) to evaluate the impact of increased reading fluency and metacognitive knowledge of reading strategies during the intervention on students’ RC. Based on these two goals, we formulated the following research questions (RQ) and hypotheses.

RQ1: What is the effect of the RT intervention on the RC of the intervention group students, both in general and students with LD, compared to the control group students’ RC after controlling for baseline RC?

*H1*: We expected the RT intervention to have a positive effect on RC in general (Schünemann et al., 2013; Gu and Lau, 2021; Wu et al., 2021) as well as in students with LD (Lee and Tsai, 2017).

RQ2: To what extent does the intervention affect the development of students’ reading fluency and metacognitive knowledge of reading strategies?

*H2a*: Intensive reading aloud practice in a certain period can improve reading fluency (Steinle et al., 2022); as the RT intervention also includes extensive practice reading aloud, we expected the reading fluency to increase during the intervention to a greater extent in the intervention group as a whole and the subgroup of students with LD than in the corresponding control groups.

*H2b*: As the RT intervention aims to teach the conscious use of reading strategies, we expected the metacognitive knowledge of reading strategies to increase during the intervention to a greater extent in the intervention group as a whole and the subgroup of students with LD (Ritchey et al., 2017; Muijselaar et al., 2018; Gu and Lau, 2021; Wu et al., 2021) than in the corresponding control groups.

RQ3: To what extent are the changes in reading fluency and metacognitive knowledge of reading strategies during the RT intervention related to students’ RC outcome when considering the basic level of RC?

*H3a*: As fluent reading and RC are strongly related (Perfetti, 2007), we expected changes in reading fluency to affect the outcome of RC to a greater extent in the intervention group.

*H3b*: As metacognition in reading helps monitor and control the process of RC more consciously (Afflerbach et al., 2020), we expected changes in the metacognitive knowledge of reading strategies to affect the outcome of RC to a greater extent in the intervention group.

## Method

### Participants

**Figure 1:**
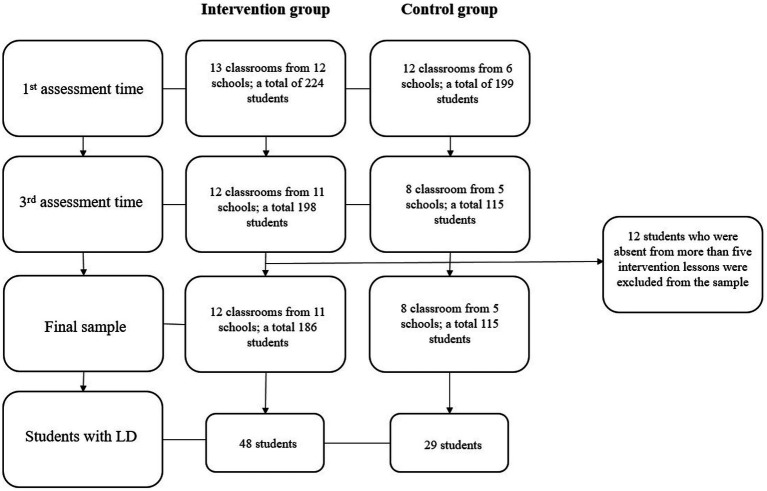
Flowchart of participants.

The Ethics Committee of Tallinn University approved this study. In this study, the sample was formed on the principle of convenience sampling. Teachers and support specialists who responded to the public advertisement and expressed willingness to participate in the intervention with their Grade 3 students were recruited to participate. An invitation was sent to participating teachers’ students (*N* = 445) and their parents, asking them to participate. Five students invited to the intervention group and 17 invited to the control group refused to participate, resulting in all participating students and their parents gave written informed consent to participate. Ultimately, 423 Grade 3 students from 18 Estonian schools participated in the first assessment. Due primarily to the Covid-19 outbreak, the size of the initial sample decreased. During the second assessment, i.e., immediately after the end of the intervention (in December 2020), it was the height of the Covid-19 outbreak in Estonia. There were many students absent during the tests and also the conditions for conducting the tests were unfavorable (e.g., the anxiety level of the students was higher). Therefore it was decided not to use the results of the second evaluation in the analysis. Thus, only the results of the first and third assessment procedure were used in the analyzes. During the third assessment, 110 students were absent. In addition, 12 students who missed more than five intervention lessons were excluded from the sample. The formation of the final sample, from the first to the third assessment, is shown in Figure 1.

In the final sample (see Figure 1), the intervention group included 186 students (50% girls, M_age_ = 9.43 years, SD = 0.39) from 11 general education schools in 12 classrooms in total (M_class size_ = 17, SD = 0.30). Of these students, 48 (25.8%) received general support at school (i.e., part-time special educational support); 18 of them were girls. The control group included 115 students (74.8% girls), whose average age was 9.41 years (SD = 0.30). Of these students, 29 (25.2%) received general support at school; nine of them were girls. Control group students were from eight classrooms total in five general education schools. The average class size was 21 students (SD = 3.37). In the present study, students who received general support at school (i.e., part-time special educational support) were considered as students with learning difficulties (LD). Students receiving enhanced or special support did not participate in the study.

In seven classes, the intervention was carried out in collaboration between the classroom teacher and the support specialist. In five classrooms, the intervention was conducted by the classroom teacher alone. Thus, a total of 12 classroom teachers and seven support specialists were involved in the intervention program; one of them was male. Of the classroom teachers, nine had a master’s degree and two had a bachelor’s degree; all of them had more than 15 years of work experience. One classroom teacher did not disclose any personal data. Of the support specialists, three were special educators and one was a speech therapist; all had a master’s degree. Three of them had more than 15 years of work experience, and one had between six and 10 years of work experience. Three support specialists did not disclose their personal data.

### Study design and content

**Figure 2:**
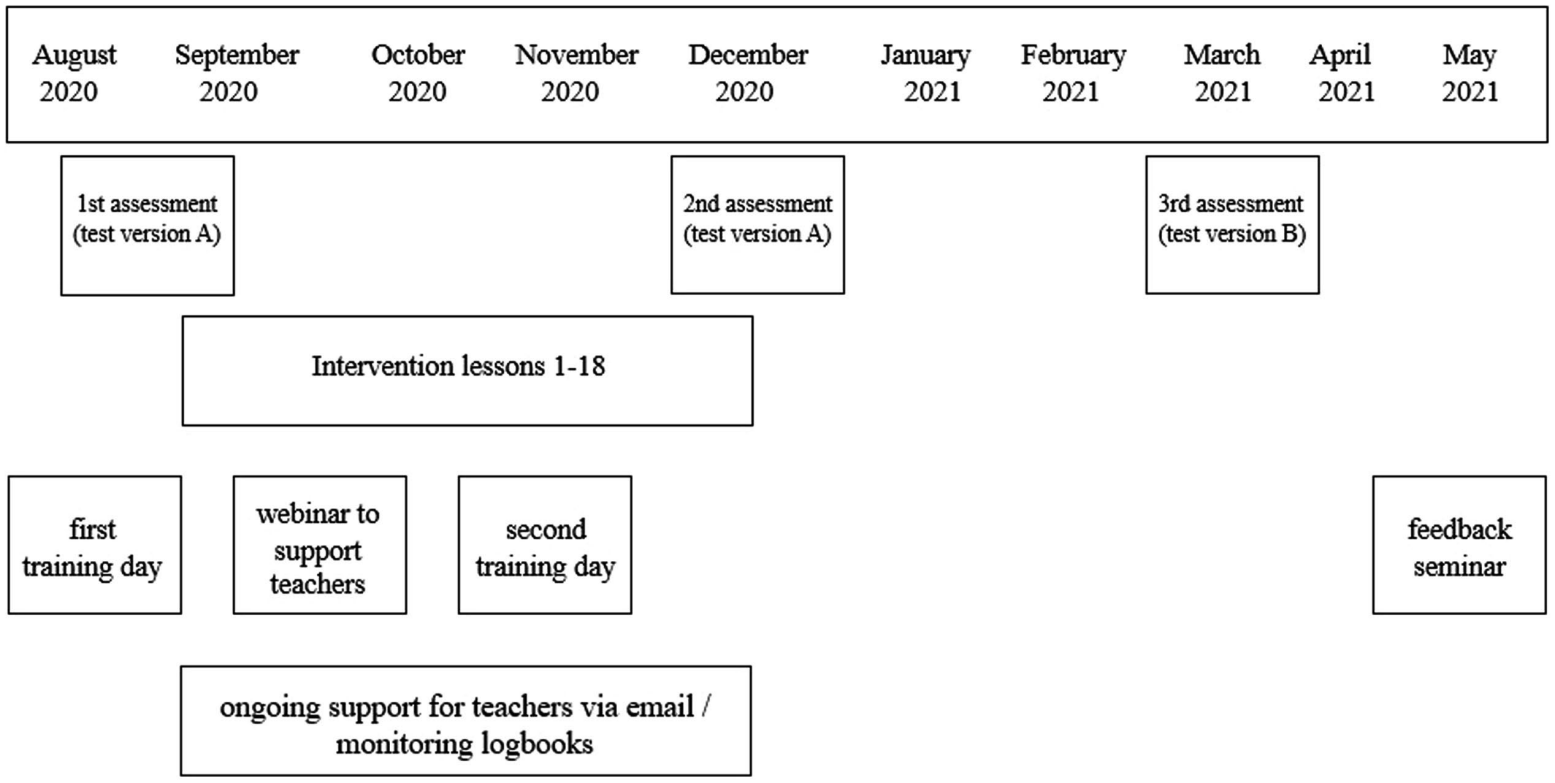
Study design and schedule.

The principles of teaching reading strategies of the intervention “We read” are based on the Reciprocal Teaching method (Palincsar and Brown, 1984) and the principles of metacognition development are based on Zimmerman’s (1998) self-regulated learning framework. The content of the intervention based on the framework of Reciprocal Teaching and self-regulated learning are presented in Table 1. Designed for primary schools, the intervention program was classroom-based and conducted by specially trained classroom teachers and support specialists. The teachers conducting the intervention were trained to follow generally accepted principles in teaching metacognitive strategies: naming the strategy and giving an explicit explanation why using this strategy is beneficial; modeling the use of a strategy by thinking aloud (i.e., explicitly teaching what to do, how to do it, and why when using a strategy, as well as explaining when this strategy could be used and when not); and continuously supporting students in reflecting on their activities and practicing the use of strategies (Kuhn and Pearsall, 1998; Kuhn et al., 2004). The intervention program consisted of 18 intervention lessons (one to two lessons per week) and lasted for 13 weeks (September 2020 to December 2020). The intervention intensity and the duration of intervention period was similar to previous studies (e.g., Spörer and Schünemann, 2014) and the performance and resources of the teachers’ conducting the intervention were also taken into account when defining the intervention intensity and duration. Intervention lessons took place during the normal school day (not as additional lessons) and were mostly integrated with Estonian language or science lessons because based on the curriculum one of the goals of these lessons is to develop students’ reading comprehension, and therefore the intervention activities were well integrated into these lessons. Both teachers and support specialists were trained before and during the intervention. Precise instructions for conducting the intervention activities were also available in teachers’ instruction materials. To monitor the implementation of the intervention according to requirements and schedule, teachers were asked to make a short entry in their personal online logbook after each intervention lesson. In the logbook, teachers added information about what text they used in the particular lesson, how much time it took to deal with the text, and what difficulties they noted (e.g., which reading strategies the students needed the most support for). Teachers also noted which students missed the intervention lesson. Researchers could access and monitor teachers’ logbooks at any time, and additional support was provided to teachers as needed. At the end of the intervention program, the researchers held a closing seminar to share the results of students’ assessment (pre- and post-test results and the impact of the intervention on skills development). More detailed information regarding the content of intervention lessons and the topics of teacher training is provided in the Appendix 1. The study design, including the schedule of intervention lessons, student assessment, and training days for teachers and support specialists, is presented in Figure 2.

**Table 1:** General content of intervention lessons.

	Teaching reading strategies through the framework of the Reciprocal Teaching method (Palincsar and Brown, 1984)	Developing metacognition through Zimmerman’s (1998) self-regulated learning framework
Lesson 1	Introducing the content and goals of the intervention. Modeling and practicing a reading strategy under the guidance of a teacher: predicting.	Goal setting and planning.
Lesson 2	Reading strategy recall: predicting. Modeling and practicing reading strategies under the guidance of a teacher: clarifying and questioning.	
Lesson 3	Reading strategies recall: predicting, clarifying, questioning. Modeling and practicing a reading strategy under the guidance of a teacher: summarizing.	Explaining the importance of goal setting and self-assessment. Setting goals for one’s own learning process before intervention activities. Assessment of one’s own learning process after the intervention activities.
Lesson 4	Introducing group work. Practicing reading strategies in a student group by monitoring and directing the learning process of oneself and others: predicting clarifying, questioning, summarizing.	Setting goals for one’s own learning process before intervention activities. Self-monitoring during the learning process. Assessment of one’s own learning process after the intervention activities.
Lessons 5–18	Practicing reading strategies in a student group by monitoring and directing the learning process of oneself and others: predicting clarifying, questioning, summarizing.	Setting goals for one’s own learning process before intervention activities. Self-monitoring during the learning process. Assessment of one’s own learning process after the intervention activities.

### Measures

An online standardized test package (Toomela et al., 2020) was used to assess students’ skills before and after the RT intervention. The assessment was the same for both the intervention and control groups. Students’ skills were assessed three times: before the intervention in September 2020 (using test version A), immediately after the intervention in December 2020 (using test version A), and 3 months after the end of the intervention in March 2021 (using test version B). The present study used the results of two assessments, the pre-intervention assessment (first assessment) and the assessment conducted 3 months after the intervention (third assessment). The tasks used are described next.

### Reading comprehension

To assess RC, students were asked to read two consecutive expository texts on the same topic. In version A of the test, the topic was the dangers of walking on weak ice and how to act in the case of danger. The two texts had a total of 358 words, with an average sentence length of 10.5 words (SD = 3.59). In version B of the test, the topic was a venomous snake in Estonia and how to act if bitten by this snake. The two texts had a total of 357 words, with an average sentence length of 11.2 words (SD = 4.81). Both test versions used the same types of tasks to assess RC: (1) sentence verification tasks (The student was shown five statements and asked to assess whether each statement was correct or incorrect based on the texts read; each correctly verified statement received one point, i.e., the maximum possible score was five points); (2) multiple choice questions (The student was displayed a question with four possible answers, one or more of which were correct, the student had to indicate all suitable answers. A maximum of four points could be obtained for one question if all correct answers were marked as correct and incorrect answers were left unmarked. There were five such questions in total, i.e., the maximum possible score was 20 points); (3) multiple choice question with five possible answers, two of which were correct (the student was allowed to mark two answers, if both were correct, the student received two points). Response time was not limited, and students were allowed to read the text again when answering. In both test version, the maximum score was 27. Based on the collected norms, the Cronbach’s alpha of the RC task in version A of the test was 0.86, and 0.83 in version B (Toomela et al., 2020).

### Metacognitive knowledge of reading strategies

To assess students’ metacognitive knowledge of reading strategies, students had to rate 12 statements that described activities as useful or not useful when reading (for example: *What is useful to do while reading? (1) I read difficult parts of the text quickly. (2) I stop reading from time to time and think about what I read. (3) I read through the last sentences, then I know how the text ends.*). The students scored one point when they rated effective strategies as useful and ineffective strategies as non-useful. All 12 statements are presented in Appendix 2. There was no time limit for completing the task. The task was the same in versions A and B (first and third assessments, respectively). The maximum raw score was 12. Based on the collected norms, the Cronbach’s alpha of the reading strategies task in version A of the test was 0.66, and also 0.66 in version B (Toomela et al., 2020).

### Word reading fluency

To assess word reading fluency, students were shown a picture and four phonologically similar words, one of which was correct; students had to click on the correct word as quickly as possible (e.g., for the picture presenting a squirrel (in Estonian, *orav*) four words were presented next to the picture: *ora* “spike,” *sorav* “fluent,” *orav* “squirrel,” *oravad* “squirrels”). Immediately after the student clicked on the word, a new image with four words was displayed. The test consisted of a total of 12 pictures (presented one by one on the screen). Different pictures and words were used in the parallel versions of the test, but the structure and difficulty of the words were similar. Both versions of the test contained eight two-syllable words, three three-syllable words and one four-syllable word. To calculate the reading fluency rate, the total time spent on the task (seconds) was divided by the number of words selected correctly (i.e., average speed in seconds of a correctly read word). If time for completing the task differed from the sample’s mean by more than three standard deviations, the reading fluency score was recoded as missing data as an outlier (affected six students from the intervention group and three students from the control group).

### Analysis strategy

When planning the study, the aim was to analyze the results of three evaluation procedures in order to determine whether, in addition to the immediate effect of the intervention (the data collected immediately after the end of the intervention; i.e., the second evaluation time), there is also a delayed effect of the intervention—that is, whether and to what extent the effect has been maintained in the months after the end of the intervention (i.e., third assessment time). As many students were absent during the second assessment due to the Covid-19 outbreak, we decided to analyze only the results obtained in the first and third assessments in this study. Analyzing these data provides valuable information about the longer-term effect of the intervention–specifically, whether and to what extent the effect of the intervention was maintained several months after the end of the intensive intervention.

Descriptives, correlations, analysis of covariance (ANCOVA), and repeated measures analysis of variance (ANOVA) were estimated using the statistical package SPSS 28.0. Path analyzes were performed in the R statistical platform (R Core Team, 2020) using package lavaan (Rosseel, 2012).

First, to analyze the effect of the intervention on students’ RC outcome, we used ANCOVA, in which the basic level of RC was set as a covariate (RQ1). Second, a repeated measure ANOVA was used to analyze the development of reading fluency and metacognitive knowledge of reading strategies in the intervention and control groups (RQ2). Third, a multi-group path analysis was applied to analyze how changes in metacognitive knowledge of reading strategies and reading fluency during the intervention explain the RC at the third time point in the intervention and control groups when considering the basic level of RC (RQ3). Corresponding change scores were calculated as follows: (1) the metacognitive knowledge change score was calculated by subtracting the first evaluation raw score from the third evaluation raw score, where a positive value showed that the level of metacognitive knowledge of reading strategies increased whereas a negative value showed that it decreased; and (2) the reading fluency change score was calculated by subtracting the first reading fluency score from the third reading fluency score, where a negative value showed that the reading fluency score decreased (i.e., the speed of reading correctly improved) and a positive value showed that it increased (i.e., the speed of reading correctly was slower). In the multi-group path analysis, all variables (RC scores, change scores of metacognitive knowledge of reading strategies and reading fluency) were used as observed variables. In the path model, gender and the status of receiving part-time special education support were used as control variables predicting all reading-related indicators. Residual variances of RC baseline score and the reading fluency and metacognitive strategies change scores were allowed to correlate. The goodness of fit level of the models was evaluated using *χ*^2^ statistic, comparative fit index (CFI), and the root mean square error of approximation (RMSEA). CFI values range between 0 and 1, with those greater than 0.90 indicating an acceptable fit. RMSEA is considered acceptable when its values are under 0.08 (Hu and Bentler, 1999).

## Results

### Descriptive statistics

Table 2 presents the means and standard deviations for the intervention group, the control group, and the subgroups of students receiving part-time special education support. Table 3 presents correlations separately for the intervention and control groups. Comparing the baseline RC of students in the intervention group and the control group we determined that the baseline levels of the groups did not differ statistically significantly, *t*(299) = 0.85, *p* = 0.396, *d* = 0.10. There were also no statistically significant differences between the groups either in baselines of reading fluency, *t*(299) = 1.01, *p* = 0.316, *d* = 0.12 or metacognitive knowledge of reading strategies, *t*(299) = 0.16, *p* = 0.871, *d* = 0.02. Comparing the baseline of RC of the intervention and control subgroups of students with LD we determined that the baseline levels of the groups did not differ statistically significantly, *t*(75) = 0.33, *p* = 0.740, *d* = 0.08. There were also no statistically significant differences between the subgroups either in baseline of reading fluency, *t*(75) = 1.06, *p* = 0.294, *d* = 0.25 or metacognitive knowledge of reading strategies, *t*(75) = 0.41, *p* = 0.684, *d* = 0.10.

**Table 2:** Descriptive statistics of the intervention group and the control group students.

Measure	Intervention group (*N* = 180–186)			Intervention group students with LD (*n* = 42–48)			Control group (*N* = 112–115)			Control group students with LD (*n* = 26–29)		
	Mean (SD)	Skewness (SE)	Kurtosis (SE)	Mean (SD)	Skewness (SE)	Kurtosis (SE)	Mean (SD)	Skewness (SE)	Kurtosis (SE)	Mean (SD)	Skewness (SE)	Kurtosis (SE)
RC (T1)	19.40 (3.80)	−0.30 (0.18)	−0.77 (0.36)	15.90 (3.50)	0.05 (0.34)	−0.86 (0.67)	19.03 (3.71)	−0.45 (0.23)	−0.29 (0.45)	15.62 (3.53)	0.24 (0.43)	−0.90 (0.85)
RC (T3)	20.63 (3.26)	−0.27 (0.18)	−0.41 (0.36)	19.67 (3.01)	−0.58 (0.34)	−0.40 (0.67)	19.70 (3.63)	−0.26 (0.23)	−0.67 (0.45)	17.38 (3.16)	−0,03 (0.43)	−0.34 (0.85)
Meta (T1)	8.23 (2.09)	−0.52 (0.18)	0.05 (3.6)	7.69 (1.70)	−0.05 (0.34)	−0.69 (0.67)	8.19 (2.04)	−0.34 (0.23)	−0.36 (0.45)	7.52 (1.88)	−0.27 (0.43)	−0.07 (0.85)
Meta (T3)	9.39 (2.00)	−0.91 (0.18)	0.40 (0.36)	8.31 (1.95)	−0.44 (0.34)	−0.24 (0.67)	9.01 (1.77)	−0.64 (0.23)	−0.18 (0.45)	8.14 (1.79)	−0.50 (0.43)	−0.17 (0.85)
Meta (Change score)	1.16 (2.36)	−0.03 (0.18)	0.68 (0.36)	0.63 (2.35)	−0.14 (0.34)	0.32 (0.67)	0.82 (1.93)	−0.18 (0.23)	1.27 (0.45)	0.62 (2.14)	−0.86 (0.43)	3.36 (0.85)
Fluency (T1)	6.56 (3.44)	1.90 (0.18)	5.17 (0.36)	9.22 (3.77)	0.39 (0.36)	2.26 (0.67)	6.36 (3.22)	1.51 (0.23)	2.65 (0.45)	8.84 (4.36)	0.71 (44)	−0.15 (0.85)
Fluency (T3)	5.05 (2.29)	2.09 (0.18)	7.16 (0.36)	6.80 (2.56)	0.39 (0.36)	3.75 (0.67)	4.88 (2.63)	2.25 (0.23)	5.60 (0.45)	7.07 (3.57)	1.54 (0.46)	0.36 (0.85)
Fluency (Change score)	−1.44 (2.32)	−0.37 (0.18)	0.33 (0.36)	−2.18 (3.00)	0.08 (0.37)	−0.63 (0.72)	−1.37 (2.59)	0.11 (0.23)	2.62 (0.45)	−1.48 (4.06)	−0.08 (0.46)	−0.11 (0.89)

RC, reading comprehension; Meta, metacognitive knowledge of reading strategies; T1, first assessment time; T3, third assessment time; students with LD, students with learning difficulties.

**Table 3:** Correlations between the variables in the intervention (above the diagonal) and control group (below the diagonal).

Measure	1	2	3	4	5	6	7	8
1. Reading comprehension (T1)	-	0.40**	0.28**	0.29**	0.00	−0.41**	−0.45**	0.10
2. Reading comprehension (T3)	0.48**	-	0.14	0.37**	0.19**	−0.24**	−0.25**	0.06
3. Meta (T1)	0.23*	0.20*	-	0.34**	−0.60**	−0.16*	−0.18*	0.02
4. Meta (T3)	0.38**	0.41**	0.49**	-	0.55**	−0.21**	−0.16*	0.13
5. Meta (Change score)	0.10	0.16	−0.60**	0.40**	-	−0.04	0.02	0.09
6. Fluency (T1)	−0.45**	−0.33*	−0.23**	−0.33**	−0.07	-	0.72**	−0.73**
7. Fluency (T2)	−0.24*	−0.24*	−0.28**	−0.43**	−0.10	0.60**	-	−0.05
8. Fluency (Change score)	0.28**	0.13	−0.07	−0.09	−0.01	−0.58**	0.30**	-

Meta, metacognitive knowledge of reading strategies; T1, first assessment time; T3, third assessment time. The range of students in the intervention group was 180–186 and in the control group 112–115. *p < 0.05. **p < 0.01.

### RT intervention effect on students’ RC

A one-way ANCOVA was conducted to examine differences between intervention and control group students on RC outcome, controlling for RC baseline. In the entire sample, we found a small effect on the RC outcome of students in the intervention group after controlling for RC baseline, *F* (1, 298) = 4.619, *p* = 0.032, η_p_^2^ = 0.015. In the subgroup of students with LD, we found a medium effect on the RC outcome, *F* (1, 74) = 10.360, *p* = 0.002, η_p_^2^ = 0.123.

### Development of reading fluency and metacognitive knowledge of reading strategies

**Figure 3:**
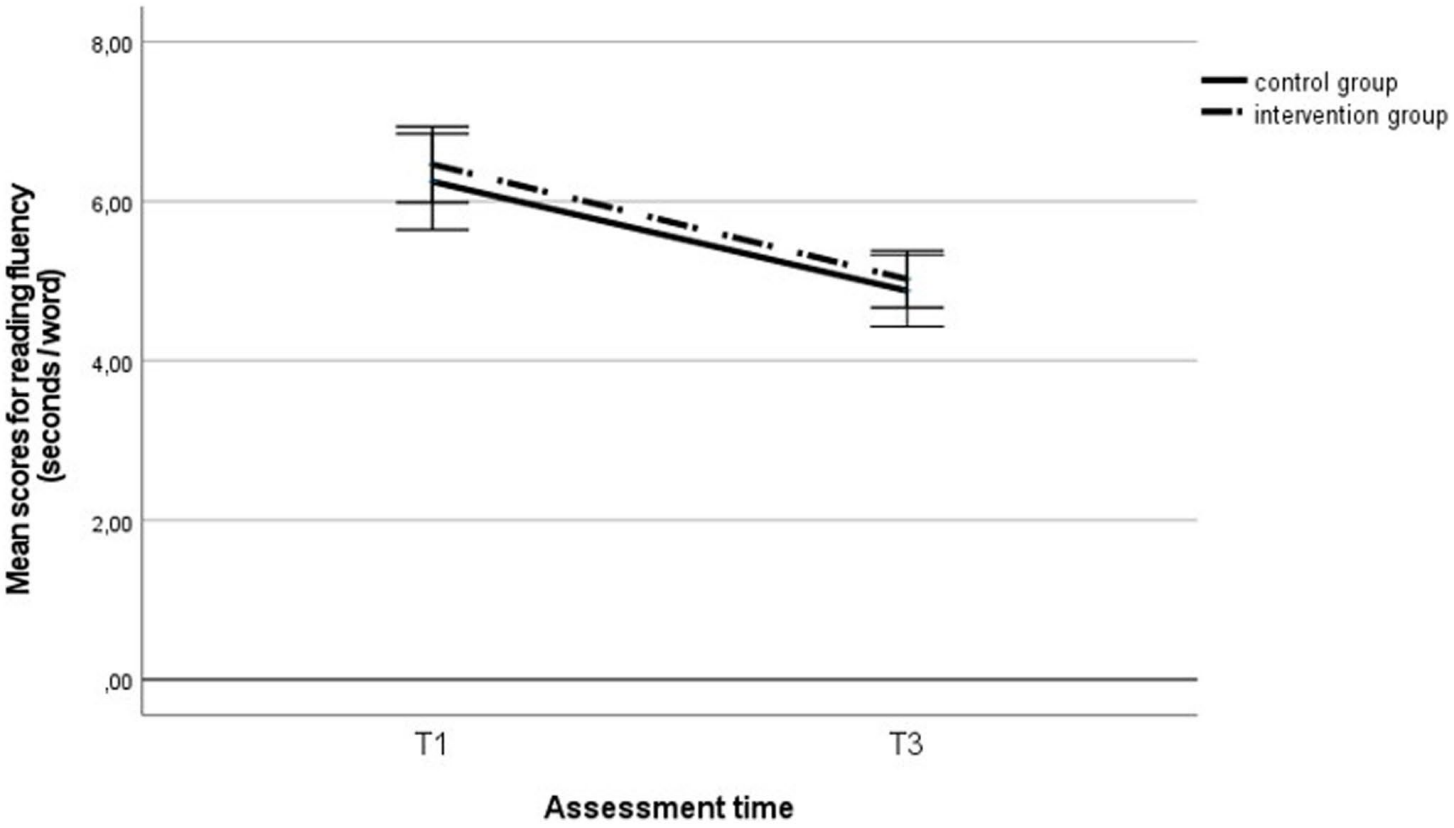
Reading fluency score in the intervention and control group students` at the first and third assessments. T1, first assessment; T3, third assessment.

**Figure 4:**
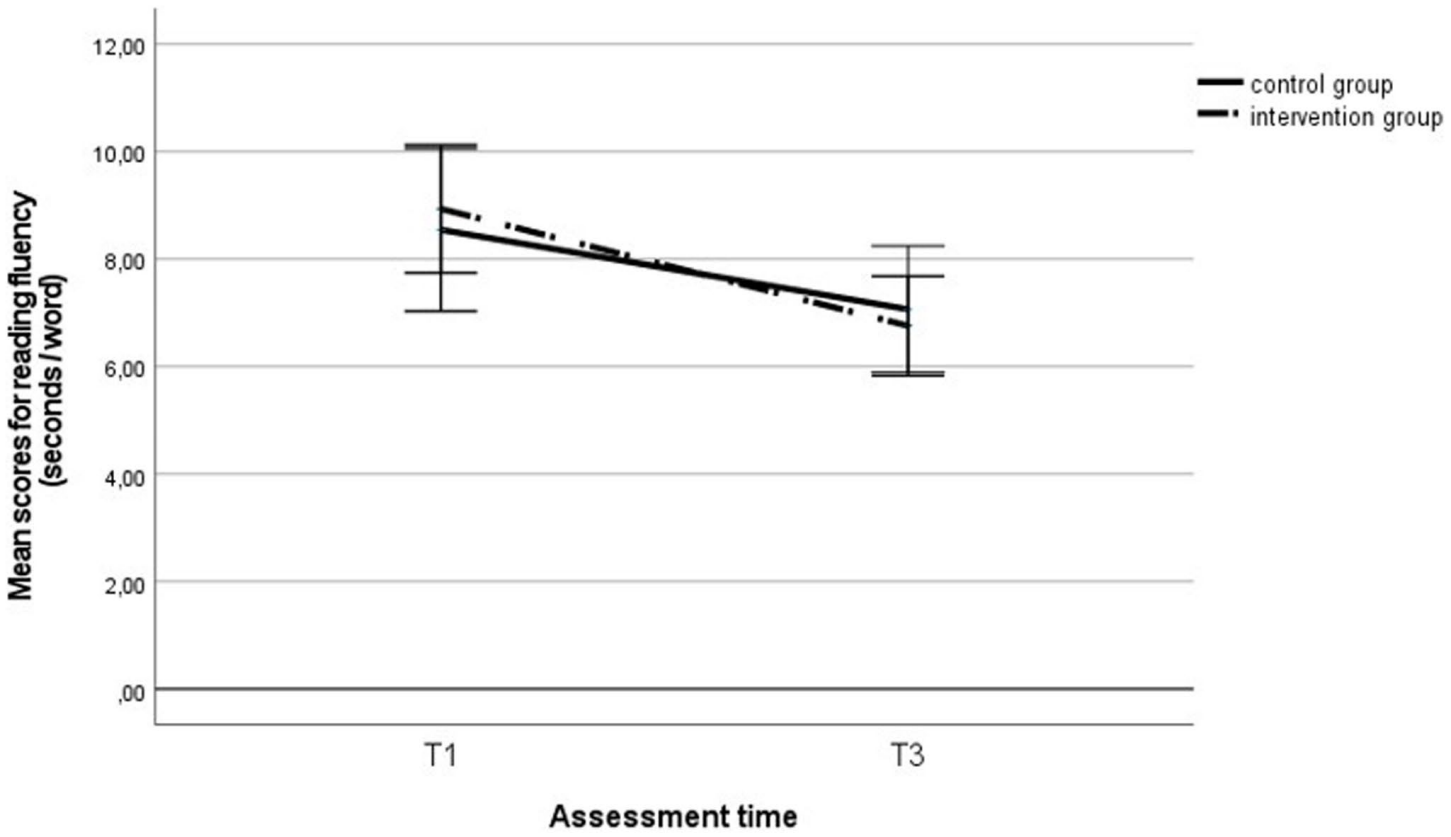
Reading fluency score in the intervention and control group students` with LD at the first and third assessments. T1, first assessment; T3, third assessment.

Using a repeated measures ANOVA, we analyzed the extent to which the reading fluency and metacognitive knowledge of reading strategies developed during the intervention period and whether belonging to the intervention group had any effect. Reading fluency improved significantly across the entire sample during the intervention, *F* (1, 290) = 92.32, *p* < 0.001, η_p_^2^ = 0.24 (see Figure 3), but no group effect was found, *F* (1, 290) = 0.34, *p* = 0.561, η_p_^2^ = 0.00, nor was there any effect of the interaction between the group and time, *F* (1, 290) = 0.06, *p* = 0.814, η_p_^2^ = 0.00. We found similar results in the subgroup of students with LD: Reading fluency improved significantly across the entire sample of students with LD, *F* (1, 66) = 18.13, *p* < 0.001, η_p_^2^ = 0.22 (see Figure 4), but no group effect was found, F (1, 66) = 0.00, *p* = 0.958, η_p_^2^ = 0.00, nor was there any effect of the interaction between the group and time, *F* (1, 66) = 0.66, *p* = 0.419, η_p_^2^ = 0.01.

**Figure 5:**
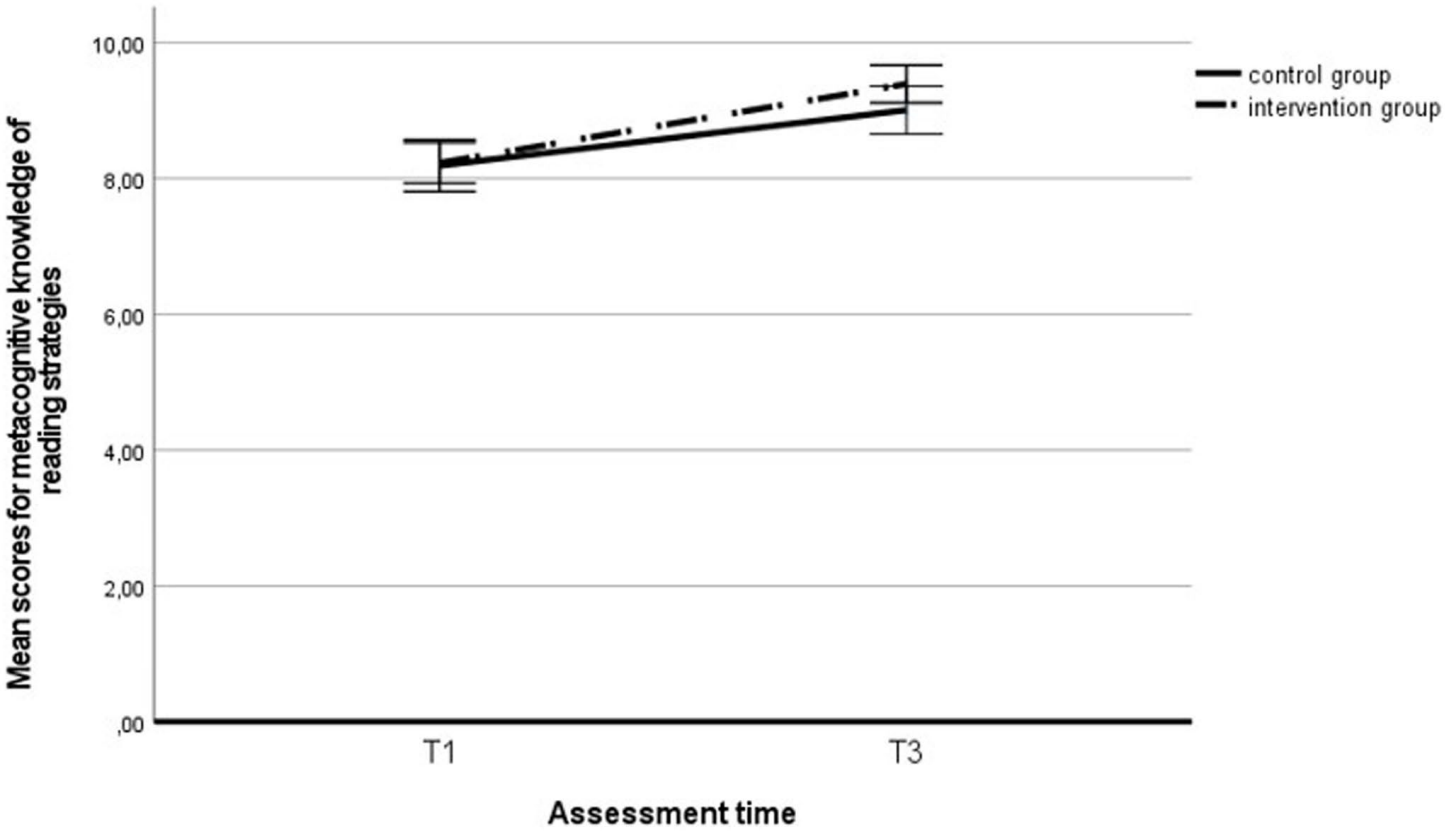
Metacognitive knowledge of reading strategies in the intervention and control group students` at the first and third assessments. T1, first assessment; T3, third assessment.

**Figure 6:**
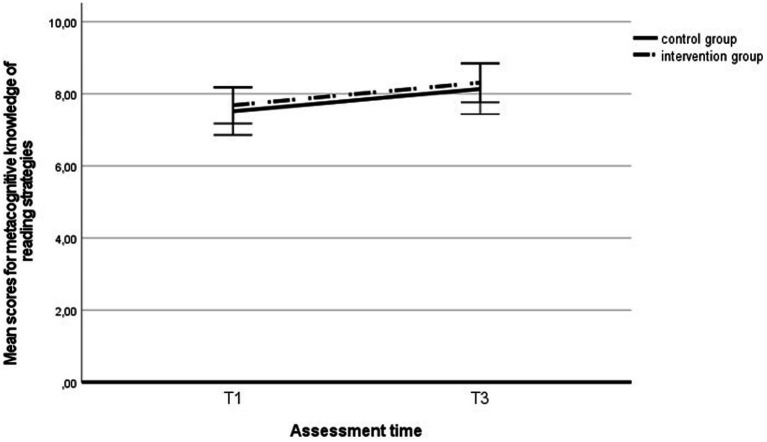
Metacognitive knowledge of reading strategies in the intervention and control group students` with LD at the first and third assessments. T1, first assessment; T3, third assessment.

Similarly, metacognitive knowledge of reading strategies increased significantly during the intervention period across the entire sample, *F* (1, 299) = 57.12, *p* < 0.001, η_p_^2^ = 0.16 (see Figure 5), whereas no difference in development was found between groups, *F* (1, 299) = 1.15, *p* = 0.284, η_p_^2^ = 0.00, nor was there any effect of the interaction between the group and time, *F* (1, 299) = 1.73, *p* = 0.190, η_p_^2^ = 0.00. We found similar results in the subgroup of students with LD: Reading fluency improved significantly across the entire sample of students with LD, *F* (1, 75) = 5.42, *p* = 0.023, η_p_^2^ = 0.07 (see Figure 6), but no group effect was found, *F* (1, 75) = 0.26, *p* = 0.611, η_p_^2^ = 0.00, nor was there any effect of the interaction between the group and time, *F* (1, 75) = 0.00, *p* = 0.994, η_p_^2^ = 0.00.

### Effect of changes in reading fluency and metacognitive knowledge on RC

To analyze the effects of changes in metacognitive knowledge of reading strategies and reading fluency during the RT intervention on students’ RC at the third assessment, considering the basic level of RC, we used a multi-group path analysis. First, we specified a model in which all parameters were unconstrained between the intervention and control groups. The model was saturated with zero degrees of freedom, which did not enable us to evaluate the goodness of model fit. However, in this model, two paths affected the RC in the third assessment differently in the intervention and control groups: The change score of metacognitive knowledge of reading strategies had a positive effect on RC outcome in the intervention group (*B* = 0.31, *SE* = 0.10, *β* = 0.22, *p* = 0.001) but a non-significant effect in the control group (*B* = 0.19, *SE* = 0.15, *β* = 0.10, *p* = 0.213); students with LD did not differ from non-LD students in RC in the third assessment in the intervention group (*B* = 0.15, *SE* = 0.56, *β* = 0.02, *p* = 0.792), while students with LD had lower RC at this time in the control group (*B* = −1.85, *SE* = 0.71, *β* = −0.22, *p* = 0.009).

**Figure 7:**
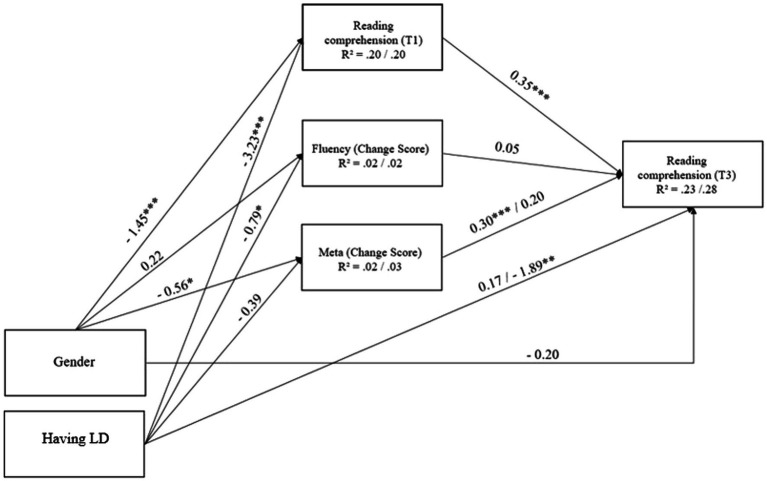
Path analysis predicting students` RC outcome in intervention and control groups. The multi-group path analysis predicts the outcome of student’s RC in the third assessments if the basic level of RC is taken into account. Unstandardized regression coefficients are presented on the figure. The number before and after the slash indicate the effects in the intervention and control groups, respectively. Meta, metacognitive knowledge of reading strategies. T1, first assessment time. T3, third assessment time. Gender is coded: 0, girl; 1, boy. Having LD is coded: 0, students without LD; 1, students with LD. *p < 0.05, **p < 0.01, ***p < 0.001.

We next compared a model with all parameters constrained between intervention and control groups and a model where two parameters, the change score of metacognitive knowledge and having LD, were unconstrained. Both models fit the data well: *χ*^2^ = 17.28, *df* = 14, *p* = 0.241, *CFI* = 0.977, *RMSEA* = 0.041 for the fully constrained model and *χ*^2^ = 11.02, *df* = 12, *p* = 0.528, *CFI* = 1.00, *RMSEA* = 0.000 for the partially constrained model. A comparison of the two models (Δ*χ*^2^ = 6.27; Δ*df* = 2; *p* = 0.044) indicated that the partially constrained model fit the data better, indicating the difference between the intervention and control groups in two unconstrained paths. Figure 7 presents the unstandardized regression coefficients of the groups (one coefficient if the parameter was constrained and two coefficients if the parameter was unconstrained); in the text, we present both unstandardized and standardized coefficients.

We first describe the effects that were fixed equal between the intervention and control groups. Girls had better RC skills at the beginning of the intervention (*B* = −1.45, *SE* = 0.41, *β* = −0.19, *p* < 0.001), but we no longer found a gender effect on the RC in the third assessment. Girls’ metacognitive knowledge of reading strategies increased more during the study (*B* = −0.56, *SE* = 0.26, *β* = −0.12 for intervention group and *β* = −0.14 for control group, *p* = 0.029), but there was no gender effect on the change of reading fluency. In both groups, students with LD had poorer RC skills in the first assessment (*B* = −3.23, *SE* = 0.45, *β* = −0.38, *p* < 0.001). The increase in reading fluency during the intervention period was also lower among students with LD (*B* = −0.79, *SE* = 0.34, *β* = −0.15 for intervention group and *β* = −0.13 for control group, *p* = 0.021). However, having LD did not affect the improvement of metacognitive knowledge of reading strategies. Students with better RC in the first assessment also showed better results in RC in the third assessment (*B* = 0.35, *SE* = 0.05, *β* = 0.40 for intervention group and *β* = 0.36 for control group, *p* < 0.001), but the reading fluency change score did not affect RC outcome in the third assessment.

Next, we describe two parameters that were unconstrained between groups. The level of metacognitive knowledge of reading strategies increased during the intervention period in both the intervention and control groups (see Figure 4), but the change affected RC in the third assessment only in the intervention group (*B* = 0.30, *SE* = 0.10, *β* = 0.22, *p* = 0.001). In the control group, the increase in metacognitive knowledge of reading strategies did not affect RC in the third assessment (*B* = 0.20, *SE* = 0.15, *β* = 0.11, *p* = 0.181). Another path that was allowed to vary was the effect of having a LD on RC in the third assessment. In the intervention group, students with LD did not have lower RC in the third assessment than non-LD students (*B* = 0.17, *SE* = 0.54, *β* = 0.02, *p* = 0.748), but in the control group students with LD still had poorer RC skills at the end of the study (*B* = −1.89, *SE* = 0.68, *β* = −0.23, *p* = 0.005). Our model explained 23% of the variance in RC in the third assessment in the intervention group and 28% of the variance in the control group.

## Discussion

The present study had two objectives: to evaluate the effect of RT intervention on students’ RC, reading fluency, and metacognitive knowledge of reading strategies and to assess to what extent the increase in reading fluency and metacognitive knowledge during the intervention affects the RC outcome. The study revealed that the intervention had a positive effect on students’ RC outcome; the effect was even stronger in the subgroup of students with LD. We also found that reading fluency and metacognitive knowledge of reading strategies improved significantly during the intervention period both in the intervention group as a whole and among students with LD, but similarly in both the intervention and control groups. Whereas the increase in reading fluency did not predict the RC outcome in either the intervention or control group, we found that the increase in metacognitive knowledge of reading strategies contributed to RC only for intervention group students, but not for control group students.

We wanted to know the effect of the intervention on students’ RC. Based on previous intervention studies (Schünemann et al., 2013; Lee and Tsai, 2017; Gu and Lau, 2021; Wu et al., 2021) we expected the intervention to have a more positive effect on RC both in the intervention group as a whole and in the intervention subgroup that included students with LD than the corresponding control groups (Hypothesis 1). The results showed that, after the intervention (during the third evaluation), a positive effect on RC was evident in the intervention group as a whole (small effect size), but especially in the intervention subgroup of students with LD (medium effect size). Thus, the results confirmed our first hypothesis, showing that the RT intervention has a positive effect on the RC of both the intervention group as a whole and students with LD. As our findings are consistent with previous meta-analyzes (e.g., Rosenshine and Meister, 1994; Lee and Tsai, 2017) as well as recent studies (Gu and Lau, 2021; Wu et al., 2021), we can again confirm that the achievement of better results in RC was probably ensured by the main focus of these kind of interventions–namely, the targeted teaching of reading strategies.

However, considering the results of the present study, it is also worth discussing the reasons that could ensure a greater effect of the intervention on the RC of intervention group students with LD. As students with LD tend to have lower cognitive abilities, attention and motivation problems (Kendeou et al., 2014; Berkeley and Larsen, 2018), and insufficient metacognitive knowledge and skills (Wigent, 2013; Bergey et al., 2017), they need a particularly explicit and concrete strategy teaching process in order to achieve success (Bosson et al., 2010). The intervention implemented in our study followed these principles (i.e., explicit teaching, modeling, and targeted practice of strategy use), which may explain why we found a greater effect on the RC of students with LD. Another possible reason to explain the effect size differences between the intervention group as a whole and the intervention group students with LD could be that many students without LD performed the tasks assessing RC quite successfully even before the intervention (i.e., the ceiling effect of the RC measurement tools must be considered). For example, approximately 40% of the students without LD got at least 80% of the answers correct in the RC tasks before the intervention (i.e., at the first assessment point), while only 12% of the students with LD achieved the same result before the intervention. This could be one explanation for the larger effect in the group of students with LD, who initially got lower scores, but whose skills to cope better with text comprehension improved during the intervention.

Second, we wanted to know the extent to which reading fluency and metacognitive knowledge of reading strategies developed during the intervention period. Based on previous studies showing that intensive reading aloud practice can have a positive effect on reading fluency (Steinle et al., 2022), we hypothesized (Hypothesis 2a) that, due to the more intensive reading aloud practice during the RT intervention, reading fluency would develop more in the intervention group than the control group. However, our results showed that in both groups students’ reading fluency improved significantly over time, with no differences between the groups. One reason why our hypothesis was not confirmed may lie in the fact that the reading speed and accuracy of Estonian Grade 3 students develops significantly over the academic year even during regular teaching; significant development also occurs among students with poorer reading skills (Jakobson et al., 2022; Juhkam et al., 2022). Thus, more intensive reading aloud practice during the intervention may not have provided enough of an advantage to students in the intervention group. We also did not have data on whether and how intensively the teachers of the control classes used reading aloud in their daily teaching practice (i.e., train reading fluency in a targeted manner); this information could probably also help interpret the results and therefore warrants more attention in future studies.

Regarding the development of metacognitive knowledge of reading strategies, we expected (Hypothesis 2b), as previous intervention studies have found (Ritchey et al., 2017; Muijselaar et al., 2018; Gu and Lau, 2021; Wu et al., 2021), that the development of metacognitive knowledge would occur faster in the intervention group. Our hypothesis was also justified by the fact that the teachers who carried out the intervention received special training in teaching strategies, and teachers’ knowledge of reading strategies has been shown to be positively related to their students’ knowledge of reading strategies (Soodla et al., 2017). Estonian teachers, without special training, generally possess rather poor knowledge of reading strategies (Jakobson et al., 2022; Juhkam et al., 2022). As expected, we found that metacognitive knowledge of reading strategies improved significantly during the intervention period, but surprisingly, the development was similar in both the intervention group and the control group as a whole as well as in the respective subgroups of students with LD. One possible explanation of the similar development of metacognitive knowledge of reading strategies in the intervention and control groups can be found in another study conducted in Estonia, in which teachers’ teaching practices were investigated (Käsper et al., 2020). Käsper et al. (2020) found that many Estonian primary school teachers reported that they use teaching strategies that encourage active learning; the emphasis is also on teaching text comprehension strategies. Thus, based on self-reports by teachers, it can be concluded that many primary school teachers teach students what to do to ensure better RC, which can, in turn, explain why the control group students’ metacognitive knowledge of reading strategies also developed. However, Käsper et al. (2020) conducted a survey study, without actually observing the lessons. Therefore, it cannot be confirmed how effectively the teachers’ applied the mentioned teaching practices and how effectively students could actually use the strategies taught to support their RC.

Third, our main aim was to determine the extent to which the increase in reading fluency and in metacognitive knowledge of reading strategies during the intervention period contributed to the RC outcome. We hypothesized that the contribution of the increased reading fluency (Hypothesis 3a) and increased metacognitive knowledge (Hypothesis 3b) to RC would be greater in the intervention group than the control group. As we previously found (RQ2), reading fluency improved significantly, suggesting that an increase in reading fluency contributed to RC outcome because good decoding skills leave more resources for dealing with RC (Perfetti, 2007). Nevertheless, in the present study, we did not find that an increase in reading fluency during the intervention significantly contributed to the RC outcome in either the intervention or control group. One explanation why Hypothesis 3a was not confirmed could be that, according to the SVR, some readers have good decoding skills but still experience insufficient comprehension (Gough and Tunmer, 1986; Hoover and Gough, 1990), which has been described as a deficiency of top-down skills. In other words, learners decode all words quickly and correctly, but their RC is still low because the meaning of many words is unknown and meaningful connections between words and sentences cannot be constructed (Catts et al., 1999; McCardle et al., 2001). Another explanation could be that, if the student has already achieved sufficient reading speed and accuracy (i.e., fluent reading), an increase in reading speed may no longer contribute to RC, and reading too fast may even decrease RC (Rayner et al., 2016).

The lack of connection between the increase in reading fluency and RC may also partly lie in the peculiarities of the measurement tool. We assessed students’ reading fluency using silent reading; students read single words instead of connected text. However, oral reading fluency and silent reading fluency are different constructs, and RC is predicted using oral reading fluency measures because they give a real picture of how quickly and correctly the student reads (Price et al., 2016). In addition, as students read silently and read words not connected text, we were unable to assess reading prosody, which is also an important component in the construct of reading fluency (Kuhn et al., 2010). Several studies have shown that prosody may be a link between fluency and RC (Veenendaal et al., 2014; Calet et al., 2015), as prosodic reading can help segment text into meaningful units based on syntactic and semantic elements; such segmentation into meaningful units supports RC (Kuhn and Stahl, 2003). Although it can be assumed that during the intervention the students paid attention to improving prosody while reading aloud, the assessment tool used in the study did not enable us to measure this effect.

In terms of metacognitive knowledge of reading strategies, we found something of key importance, which can also explained the previously raised issue regarding the equal increase in metacognitive knowledge of students in the intervention and control group. Although metacognitive knowledge of reading strategies increased equally in both groups, the increase in metacognitive knowledge still predicted RC, but only in the intervention group. Thus, Hypothesis 3b, which assumed that an increase in metacognitive knowledge of reading strategies contributes to the RC outcome to a greater extent among students in the intervention group, was confirmed. The explanation for this result probably lies in the process of teaching metacognitive knowledge and skills. When teaching reading strategies, as in the process of teaching all metacognitive knowledge and skills, learners’ declarative, procedural, and conditional knowledge must be ensured because only in this way can the use of new knowledge and skills be effective (Flavell, 1979; Brown, 1987). Based on our finding, it can be concluded that the intervention provided good knowledge about effective reading strategies (declarative knowledge) as well as the ability to use strategies in a purposeful way (procedural and conditional knowledge), which is likely why we found that the increase in metacognitive knowledge in the intervention group actually contributed to RC. Otherwise, if all aspects are not taught sufficiently, it may happen, for example, that the student knows effective reading strategies (i.e., has declarative knowledge), but does not use these strategies while reading (i.e., lacks procedural knowledge). This issue likely appeared in the control group and explains why the metacognitive knowledge of reading strategies increased among control group students, but did not contribute to their RC. Therefore, this study may be an indication that, without special training, teachers’ competences in teaching reading strategies may be insufficient. This argument is also supported by earlier observational studies that found when teachers without special training teach reading strategies, they did so in narrow and mostly indirect ways (Anmarkrud and Bråten, 2012), and teachers usually spent quite a bit of time teaching reading strategies (Barron et al., 2018). However, the effective teaching of reading strategies requires quite the opposite approach—that is, explicit instruction (Pressley and Gaskins, 2006) and enough time for guided practice (Houtveen and van de Grift, 2007). Thus, teacher training is crucial to ensure that teachers have all the necessary knowledge in the learning process of using reading strategies and can achieve the main goal: knowing and using reading strategies so that they actually contribute to RC.

### Limitations and conclusion

Some limitations must also be taken into account when interpreting our results. First, the limitations of the measurement tools used in the study must be emphasized. The results may have been affected by the fact that the RC assessment instrument may have had a ceiling effect in our sample; therefore, we may not have captured the actual development of students’ RC, especially among more skilled students. In addition, as argued in the discussion section, silent word reading may not have measured those indicators of reading fluency that could actually contribute to RC. Second, the fidelity aspect of the study must be emphasized. Although we monitored teachers’ implementation of the intervention by continuously observing the entries in the online logbooks, we did not observe the intervention lessons; therefore, we do not really have an overview of how well and precisely the teachers followed the intervention instructions. In addition, the results may have been influenced by the fact that, in some classes, the teachers implemented the intervention in isolation whereas others classes implemented it in collaboration with a support specialist. Although the comparison of subgroups of the intervention was not the focus of our research, this aspect should be investigated more precisely in order to confirm whether its implementation in collaborative work could have a greater effect on the RC of students with LD. Third, it must be emphasized that analyzing the results of the second assessment time would have added value to the study, but due to the Covid-19 outbreak, conducting the tests in the second assessment time was complicated, and therefore it was decided to exclude the second assessment time from the analysis.

The results of the present study confirm that RT intervention to improve RC is effective, especially for students with LD. Furthermore, an important concern when teaching reading strategies and, thus, supporting RC is that the process of teaching reading strategies must be accomplished not only by providing declarative knowledge of strategies, but also by supporting the acquisition of procedural knowledge of the effective use of strategies. Only in this way is it possible to use the learned strategies effectively while reading (i.e., in such a way that they actually contribute to RC). Therefore, adopting interventions in which teachers are purposefully trained to ensure a deep knowledge of the effective learning process in teaching metacognitive knowledge and skills to both pre-service and in-service teachers can be key in supporting the development of students’ RC.

## Data availability statement

The raw data supporting the conclusions of this article will be made available by the authors, without undue reservation.

## Author contributions

MJ contributed to the development of the intervention program and the implementation of the intervention, contributed to data collection and analysis, and wrote the first draft of the manuscript. A-LJ contributed to data analysis and manuscript revision. PS contributed to the development and implementation of the intervention program and manuscript revision. MA contributed to the manuscript revision. All authors contributed to the article and approved the submitted version.

## Conflict of interest

The authors declare that the research was conducted in the absence of any commercial or financial relationships that could be construed as a potential conflict of interest.

## Publisher’s note

All claims expressed in this article are solely those of the authors and do not necessarily represent those of their affiliated organizations, or those of the publisher, the editors and the reviewers. Any product that may be evaluated in this article, or claim that may be made by its manufacturer, is not guaranteed or endorsed by the publisher.
