# Supplementary material for: Development of reading fluency and metacognitive knowledge of reading strategies during reciprocal teaching: do these changes actually contribute to reading comprehension?
Source: Front Psychol. 2023 Aug 24;14:1191103. doi: 10.3389/fpsyg.2023.1191103 (PMC10502224; doi:10.3389/fpsyg.2023.1191103)
Supplement: Supplementary file 1 [file Table_1.docx]

**Appendix 1**

**Schedule and content of the “We Read” intervention program**

When the teacher and the support specialist implemented the program together, some of the intervention lessons were conducted in the same classroom with all students while other intervention lessons were conducted separately (i.e., the teacher worked in the classroom and the support specialist worked with students having difficulty in a smaller group in a separate room to support them more). The structure and content of the lessons conducted separately by the teacher and the support specialist were the same.

One lesson is about 45 minutes. The texts used in the lessons consist of four passages.

| **Time** | **Classroom teacher** | **Support specialist** |
| --- | --- | --- |
| **1. Training day** *(8 academic hours)*  Content: the nature and development of reading skills; reading strategies; the theoretical basis of the “We Read” intervention program; practical exercises for implementing the intervention program. | | |
| Lesson 1 | Lesson content:  ● The teacher discusses with the students why good reading skills are necessary (e.g., good reading skills help them learn better).  ● The teacher introduces the "We Read" program to the students (e.g., the purpose of the program is to help students become better readers; the duration of the program is 18 lessons).   - Introducing the first reading strategy: prediction - Students read the title of a text together with the teacher, and after reading the title, the teacher models the prediction strategy and predicts what the text could be about based on the title. The teacher also explains, thinking aloud, what she does during predicting and how predictions supports reading comprehension (e.g., activation of prior knowledge). - The students also make predictions based on the title. Next, the first part of the text is read to check whether the predictions were accurate. - The teacher makes a new prediction based on the part of the text already read (i.e., predicts what the text could talk about next). Students also make their own predictions. The next part of the text is then read to check whether the predictions were accurate. - Similar work is done with all four text passages.   ● At the end of the lesson, the teacher summarizes the lesson, once again emphasizing why making predictions is important and how predictions support reading comprehension.  *The lesson is conducted together.* | |
| Lesson 2 | Lesson content:  ● Recalling what was learned in the previous lesson (i.e., prediction strategy)   - Introducing the second and third reading strategies: clarifying and questioning - Make predictions based on the title of the text and then read the first part of the text to check the predictions. - The teacher then directs students’ attention to the words/expressions that are difficult in this part of the text and models by thinking aloud what to do to understand the difficult words/expressions (e.g., I read the sentence again; I read a little further and try to find clues in the text that help me understand the difficult word/expression). - The teacher also explains why using the clarifying strategy is important—namely, it helps monitor reading comprehension while reading and, if necessary (i.e., if we notice that we do not understand what we read), find ways to ensure reading comprehension. - The students look for difficult words/expressions in that part of the text and talk about what they do to understand the meaning of these words/expressions and clarify them. - Once the work of clarification is done, the teacher models the questioning strategy. The teacher asks questions about the read passage (using different question words; e.g., *Who? Where? When? Why?*) and discusses aloud whether the answers to the given questions can be found directly in the text or whether information must be derived and inferred to get the answer. - The teacher explains why asking questions is necessary and how it supports reading comprehension (e.g., it helps check whether I have understood the text, whether I can ask questions about important information). - Students ask and answer questions about the passage they read. - Students move on to the next text passage, and the whole process is repeated: before reading, predictions are made, then the text passage is read and predictions are checked, then difficult words/expressions are searched for and students try to explain them, and finally questions are asked about the text passage. - Similar work is done with all four text passages.   ● At the end of the lesson, the teacher summarizes the lesson, emphasizing why predicting, clarifying, and questioning are important when reading and how these strategies support reading comprehension.  *The lesson is conducted together.* | |
| Lesson 3 | Lesson content:  ● Recalling what was learned in the previous lesson (i.e., predicting, clarifying, questioning)   - Introducing the fourth reading strategy: summarizing - The title of the text and the first passage of the text are read, and the strategies of prediction, clarifying, and questioning are used (see previous lesson content). - Once the work of predicting, clarifying, and questioning is done with the first passage of the text, the teacher models the strategy of summarizing and explains by thinking aloud what is important in summarizing (e.g., I emphasize the most important/interesting information in the summary). The teacher also explains how summarizing supports reading comprehension (e.g., it helps us think about what we have read, check if we can find the most important information and convey it in our own words). - The students try to summarize the content of the passage they read in their own words. - In a similar way, the work continues with the following text passages (predicting, clarifying, questioning, summarizing).   ● In this lesson, the teacher also introduces students to the statements on the self-assessment sheets (setting goals before doing the reading task, such as "How well do I want to do the reading task today?", and self-evaluation after doing the reading task, such as "How well did I do in reading today?"). In subsequent lessons, self-assessment sheets will be completed both before and after reading the text.   - At the end of the lesson, a summary of the lesson is made, during which the teacher emphasizes why predicting, clarifying, questioning, and summarizing are important during reading and how these strategies support reading comprehension.   *The lesson is conducted together.* | |
| Lesson 4 | Lesson content:  ● At the beginning of the lesson, groups of students are formed (mostly 4 members). The teacher explains that students will now practice the learned reading strategies in groups, supporting each other.  ● After reading the title of the text, all students individually complete the self-assessment sheet (goal setting).  ● Students read the text in groups using the four strategies: Before reading the passage, predicting is used; after reading the passage, clarifying, questioning, and summarizing are used. Students read the whole text following the same process.  ● After reading the text, students fill in the self-assessment sheet again to evaluate how well they coped with the reading task today.  - During the lesson, the teacher supports students as needed (e.g., modelling strategies).  *The teacher works in the classroom.* | *The support specialist works with students having difficulties (in a smaller group in a separate room).* |
| Lesson 5 | Lesson content:  The same content as in Lesson 4.  *The teacher works in the classroom.* | *The support specialist works with students having difficulties (in a smaller group in a separate room).* |
| **Webinar** *(2 academic hours)*  Content: gathering initial feedback from teachers, including teacher guidance, motivation, and encouragement | | |
| Lessons 6 and 7 | Lesson content:  ● The same content as in Lesson 4.  *The lessons are conducted together.* | |
| **2. Training day** *(6 academic hours)*  Content: supporting student motivation; additional techniques for practicing reading strategies and group work; selection and adaptation of texts  **Task 1** for teachers: Assess students’ ability to use reading strategies, the level of text comprehension, and group work skills. | | |
| Lessons 8 and 9 | Lesson content:  ● The same content as in Lesson 4.  *The lesson is conducted together.* | |
| Lessons 10 and 11 | Lesson content:  ● The same content as in Lesson 4.  The teacher analyzes and reflects on the lessons in writing (choose either the 10^th^ or 11^th^ lesson). | Lesson observation by a support specialist (choose either the 10^th^ or 11^th^ lesson). |
| **Task 2** for teachers: Conduct a written analysis and reflection on the lesson. | | |
| Lesson 12 | Lesson content:  ● The same content as in Lesson 4.  *The teacher works in the classroom.* | *The support specialist works with students having difficulties (in a smaller group in a separate room).* |
| Lessons 13–18 | Lesson content:  ● The same content as in Lesson 4.  *The lesson is conducted together or separately, depending on the needs of the students.* | *The lesson is conducted together or separately, depending on the needs of the students.* |
| **Task 3** for teachers: Assess students’ ability to use reading strategies, the level of text comprehension, and group work skills; write an analysis of the development of the class as a whole and of the individual development of two students. | | |
