# Supplementary material for: Development of reading fluency and metacognitive knowledge of reading strategies during reciprocal teaching: do these changes actually contribute to reading comprehension?
Source: Front Psychol. 2023 Aug 24;14:1191103. doi: 10.3389/fpsyg.2023.1191103 (PMC10502224; doi:10.3389/fpsyg.2023.1191103)
Supplement: Supplementary file 2 [file Table_2.DOCX]

**Appendix 2**

Statements about effective and ineffective reading strategies.

What is useful to do before reading?

1. I think about what I already know about this topic. (effective)

2. I read the last sentence, then I know how the story ends. (ineffective)

3. I wonder what this text could be about. (effective)

What is useful to do while reading?

1. I read difficult parts quickly. (ineffective)

2. I stop reading from time to time and then and think about what I read. (effective)

3. I read the last sentence, then I know how the story ends. (ineffective)

What is useful to do after reading?

1. I ask myself questions about what I read and answer them. (effective)

2. I check if I have read this text before. (ineffective)

3. I count how many pages I read without making reading mistakes

What is useful if I don't understand a word or a sentence?

1. I check if this word is explained in the text. (effective)

2. I look up the meanings of the difficult words in this sentence in the dictionary. (effective)

3. I will skip this place. (ineffective)
